# Activation-induced cytidine deaminase causes recurrent splicing mutations in diffuse large B-cell lymphoma

**DOI:** 10.1186/s12943-024-01960-w

**Published:** 2024-02-24

**Authors:** Maria S. Benitez-Cantos, Carlos Cano, Marta Cuadros, Pedro P. Medina

**Affiliations:** 1 GENYO. Centre for Genomics and Oncological Research: Pfizer / University of Granada / Andalusian Regional Government, PTS Granada - Avenida de la Ilustración 114, Granada, 18016 Spain; 2 Health Research Institute of Granada (Ibs.Granada), Avenida de Madrid 15, Granada, 18012 Spain; 3 Department of Biochemistry and Molecular Biology III and Immunology, Faculty of Medicine, University of Granada, Avenida de la Investigación 11, Granada, 18016 Spain; 4 Department of Computer Science and Artificial Intelligence, School of Computer and Telecommunication Engineering, University of Granada, Calle Periodista Daniel Saucedo Aranda s/n, Granada, 18014 Spain; 5 Department of Biochemistry and Molecular Biology I, Faculty of Sciences, University of Granada, Avenida de Fuentenueva s/n, Granada, 18071 Spain

**Keywords:** B-cell lymphoma, Somatic hypermutation, Splicing mutations

## Abstract

Diffuse large B-cell lymphoma (DLBCL) is the most common lymphoma. A major mutagenic process in DLBCL is aberrant somatic hypermutation (aSHM) by activation-induced cytidine deaminase (AID), which occurs preferentially at RCH/TW sequence motifs proximal to transcription start sites. Splice sequences are highly conserved, rich in RCH/TW motifs, and recurrently mutated in DLBCL. Therefore, we hypothesized that aSHM may cause recurrent splicing mutations in DLBCL. In a meta-cohort of > 1,800 DLBCLs, we found that 77.5% of splicing mutations in 29 recurrently mutated genes followed aSHM patterns. In addition, in whole-genome sequencing (WGS) data from 153 DLBCLs, proximal mutations in splice sequences, especially in donors, were significantly enriched in RCH/TW motifs (*p* < 0.01). We validated this enrichment in two additional DLBCL cohorts (N > 2,000; *p* < 0.0001) and confirmed its absence in 12 cancer types without aSHM (N > 6,300). Comparing sequencing data from mouse models with and without AID activity showed that the splice donor sequences were the top genomic feature enriched in AID-induced mutations (*p* < 0.0001). Finally, we observed that most AID-related splice site mutations are clonal within a sample, indicating that aSHM may cause early loss-of-function events in lymphomagenesis. Overall, these findings support that AID causes an overrepresentation of clonal splicing mutations in DLBCL.

**Graphical Abstract:**

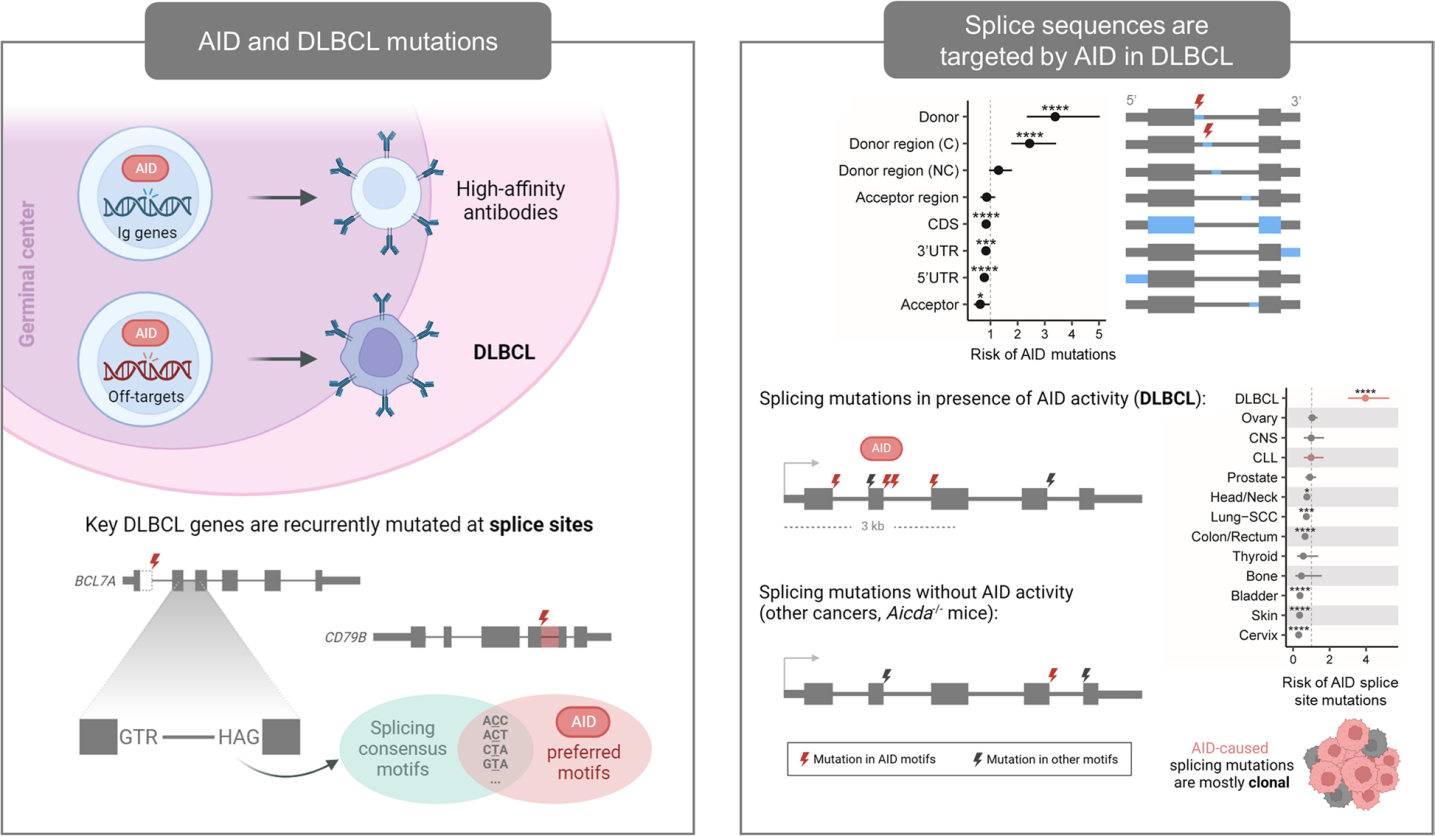

**Supplementary Information:**

The online version contains supplementary material available at 10.1186/s12943-024-01960-w.

## Introduction

Diffuse large B-cell lymphoma (DLBCL) is the most common lymphoid malignancy [1]. The high heterogeneity of DLBCL is recently being deciphered, resulting in novel classification systems based on specific genetic alterations [2]. One major mechanism of mutagenesis in DLBCL is aberrant somatic hypermutation (aSHM) caused by off-target effects of the activation-induced cytidine deaminase (AID) enzyme during the germinal center reaction [3]. According to mutational signatures studies in DLBCL samples [4–7], AID causes C > T transitions in RCH (R: A or G; H: not G) sequence contexts in single-stranded DNA (usually in transcription bubbles). As a result, aSHM-related mutations tend to be clustered within a window of up to ~ 2.5–3 kb downstream of transcription start sites (TSSs) [4, 8], especially in genes that are highly expressed in germinal center B-cells. Moreover, errors in the repair of AID-caused deaminations can generate other types of mutations [9]. First, errors in base excision repair mediated by the uracil-DNA glycosilase (UNG) can create any type of substitution at RCH sites. In addition, mismatch repair mechanisms mediated by the mutS homologs 2 and 6 (MSH2/MSH6) sometimes repair the C > T transitions caused by AID, but introduce substitutions in nearby TW contexts (W: A or T).

**Fig. 1:**
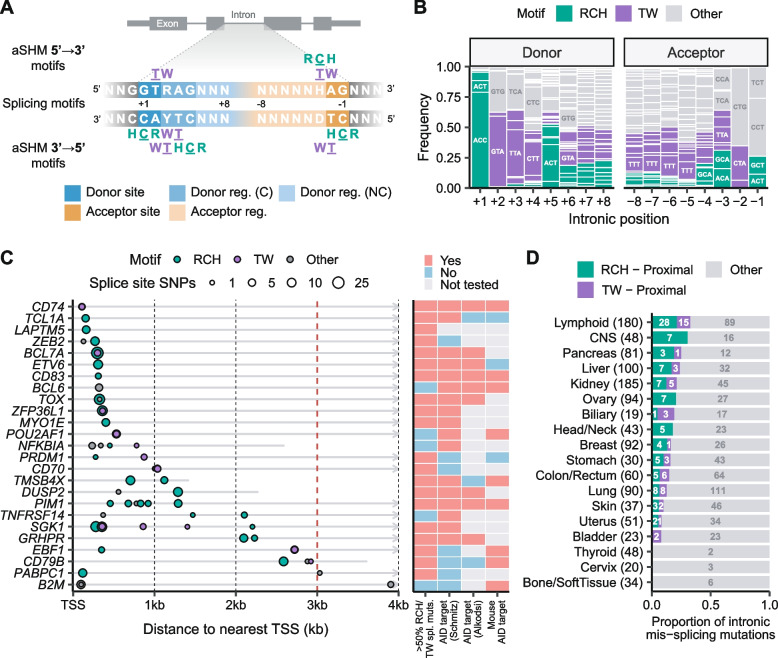
Splice mutations in DLBCL and their relationship with aSHM. **A** RCH (R: A or G; H: not G) and TW (W: A or T) motifs within splicing consensus sequences. Splice sequences can be divided into splice sites (positions ± 1 and ± 2) and splice regions (positions ± 3 to ± 8). C: conserved; NC: non-conserved. **B** Proportion of RCH and TW motifs across the human genome for each splice sequence position. **C** Recurrent splice site mutations in DLBCL from Andrades et al. [15] and their distance to the nearest TSS. Circle color represents the nucleotide context and size indicates mutation frequency. Grey lines show transcript length, with transcripts exceeding the plot limits represented by arrowed lines. The chosen threshold to classify mutations into proximal (< 3 kb) or distal (> 3 kb) is marked with a red dashed line. 4 out of the 29 genes described by Andrades (*FAS*, *KMT2D*, *TBL1XR1* and *TNFAIP3*) have been omitted for visualization purposes as their splice site mutations far exceed the 4 kb plot limit. The heatmap shows the association of each gene to AID mutagenesis. > 50% RCH/TW splicing mutations, the splice site mutations are mostly in RCH or TW contexts; AID target, the gene has been reported as an AID target by Schmitz et al. [1], Alkodsi et al. [4] or Álvarez-Prado et al. [16]. **D** Proportion of proximal RCH and TW intronic mis-splicing mutations (in positions ± 1 to ± 8) per cancer type described by Jung et al. [12]. Sample sizes are indicated in parentheses, number of mis-splicing mutations with each sequence motif are indicated in the bars. CNS: central nervous system

Splicing is a process by which introns of primary transcripts are removed and exons are joined together. Correct splicing is essential to generate functional gene products, and therefore the boundaries between exons and introns are well-delimited by highly conserved sequences [10]. The most conserved positions are the first and last two intronic nucleotides, known as splice donor and acceptor sites, respectively (Fig. 1A). Other intronic nucleotides are also highly conserved, especially at the third and fifth donor positions. Sequence changes in any of these conserved nucleotides can cause significant aberrations in gene products and are frequent events selected in cancer development [11, 12]. Aberrant spliced transcripts in most cases result in protein loss-of-function due to the appearance of a premature stop codon in the reading frame, a phenomenon observed in many cancer types particularly affecting tumor suppressor genes [12]. Moreover, tumors exhibit about a 20% increase in alternative splicing events compared with normal samples [13], which can also contribute to the generation of neoantigens that influence the immunogenicity of the tumor [14]. Recently, we reanalyzed a meta-cohort of > 1,800 DLBCLs and identified 29 genes that were recurrently mutated at their splice sites, highlighting the importance of splice site mutations in lymphomagenesis [15].

The splice donor and acceptor consensus sequences contain various RCH and TW motifs [10] (Fig. 1A), leading us to hypothesize that aSHM may be a major source of mutations in intronic splice sequences in DLBCL. Notably, 96.9% nucleotides in the splice donor position + 1 are RCH, and > 60% of the other conserved positions in splice donor sites (+ 2) and regions (+ 3, and + 5) contain RCH/TW motifs (Fig. 1B). The WRCH motif derived from studies of AID targets on immunoglobulin genes [17] is also conserved for the + 1 position of the donor (Fig. 1A) and moderately also for the -3 position in the acceptor, the latter due to the functional polypyrimidine tract located upstream of the acceptor site (Fig. 1B). Indeed, we previously showed that the tumor suppressor gene *BCL7A*, a member of the SWI/SNF complex [18], is recurrently mutated at its first splice donor site in DLBCL and that these mutations are likely caused by AID [19]. We also described the role of the mutations in the fourth donor splice site of *CD79B* [15], a gene encoding a B cell receptor accessory protein that has been found to be a target of aSHM with a bimodal distribution in DLBCL [8]. Here, we explore whether these observations can be extended to other DLBCL genes, and to what extent the putative enrichment of aSHM-related splice mutations in DLBCL can be explained by preferential mutation of AID at splice sequences.

## Methods

Somatic mutations from 3 DLBCL cohorts and 12 other cancer types (Additional file 1) were reannotated to study the enrichment in splice mutations in lymphoid malignancies with AID activity. The trinucleotide context of each variant was retrieved and mutations were considered to be proximal to a TSS when located within 3 kb, and distal when located beyond 3 kb (Fig. 1C). Single base substitutions in RCH or TW contexts proximal to a TSS were considered to follow an aSHM pattern. The distribution of mutations in aSHM/non-aSHM contexts in a given genomic feature was compared to that of intronic mutations for whole-genome sequencing (WGS) datasets or to the proportion of aSHM contexts observed in the reference genome in that feature for WGS and whole-exome sequencing (WXS) datasets. Targeted DNA sequencing data from Peyer’s patches germinal center B-cells of *Aicda*^−/−^ and *Ung*^−/−^*Msh2*^−/−^ mice [16] were reanalyzed to calculate the C > T transition frequency per genomic feature. For detailed procedures, see Supplemental text file 1.

## Results and discussion

First, we re-explored our previously identified 29 genes recurrently mutated at splice sites in over 1,800 DLBCLs to test whether their mutations may be predominantly caused by aSHM [15] (Fig. 1C). Over the 29 genes, we found that 245 (77.5%) of their mutations were consistent with aSHM patterns (in RCH/TW motifs and within 3 kb from the TSS). In addition, for 20/29 (69%) genes, the majority of splice site mutations were consistent with aSHM. Our observations agreed with previous reports. For example, Schmitz et al. [1] reported aSHM target predictions for 28 of our candidate genes, out of which 17 (61%) were significant. Alkodsi et al. [4] identified 9/12 (75%) as targets of an “RCH” mutational signature in a meta-cohort of DLBCLs. Furthermore, Álvarez-Prado et al. [16] experimentally identified 10/14 (71%) of our candidate genes as AID off-targets in mice. Moreover, intronic mis-splicing mutations (positions ± 1 to ± 8) identified by Jung et al. [12] in the International Cancer Genome Consortium (ICGC) German non-Hodgkin lymphoma cohort (MALY-DE) are the most enriched in proximal RCH/TW motifs over all analyzed cancer types (Fig. 1D). Taken together, these observations suggest that recurrent splice mutations in DLBCL are associated with aSHM.

**Fig. 2:**
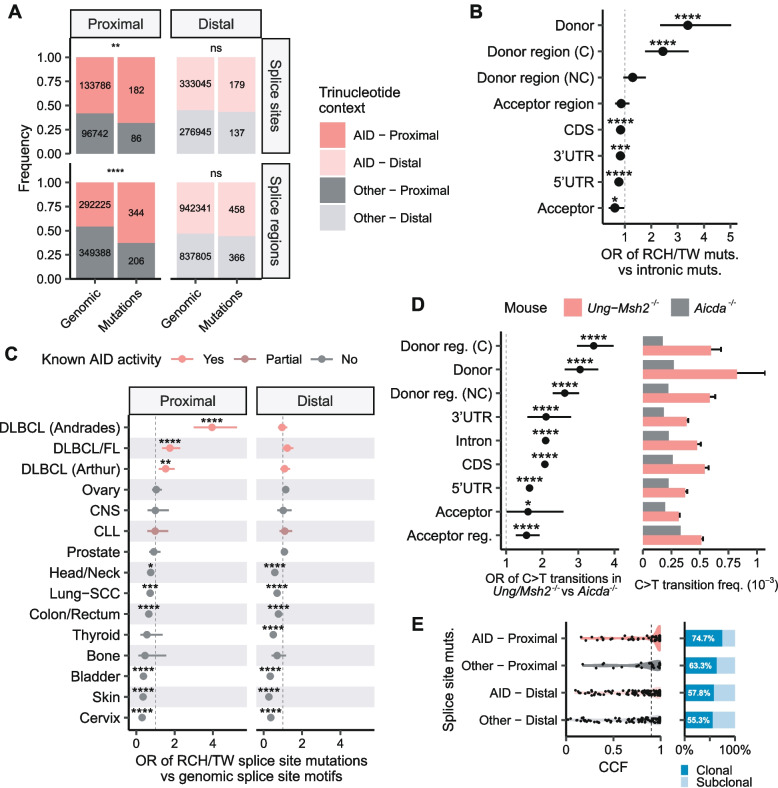
Proximal splice mutations are enriched in aSHM motifs in DLBCL. **A** Proximal splice site and splice region mutations in DLBCL are significantly enriched in AID motifs compared with the motif distribution of all splice sites and regions annotated in the human genome. **B** Enrichment analysis of proximal RCH and TW mutations in each genomic feature compared with proximal intronic mutations. **C** Pan-cancer enrichment analysis of splice site mutations at RCH or TW motifs compared with the motif distribution of all splice sites annotated in the reference genome. Color indicates whether AID-related mutational signatures have been found in a cancer type. “Partial” indicates that the AID activity was present in less than 50% of the samples analyzed [21]. FL: follicular lymphoma; CLL: chronic lymphocytic leukemia; CNS: central nervous system; SCC: squamous cell carcinoma. **D** Enrichment in G/C transition frequency per genomic feature in *Ung*^−/−^*Msh2*^−/−^ mice (N = 2) compared with *Aicda*^*−/−*^ mouse (N = 1). C: conserved; NC: non-conserved; CDS: coding sequence; UTR: untranslated region; OR: odds-ratio. In all panels, Fisher’s exact test FDR-corrected p values are shown (ns: non-significant; *: *p* < 0.05; **: *p* < 0.01; *** *p* < 0.001; **** *p* < 0.0001). **E** Estimated cancer cell fraction (CCF) distributions of splice site mutations from Chapuy et al. DLBCL cohort [6]. Mutations are divided into four categories regarding their nucleotide context and their distance to the nearest TSS. A variant is considered clonal when its CCF ≥ 0.9 (dashed line), the proportion of clonal and subclonal mutations in each category is showed in the bar plot

Next, we wondered if DLBCLs are enriched in mutations at aSHM motifs in splice sites (intronic positions ± 1 and ± 2) or splice regions (intronic positions ± 3 to ± 8) over other genomic features. To this end, we reanalyzed the WGS dataset of Arthur et al. [20]. In a first approach, as a background distribution, we considered the proportion of aSHM motifs in the splice sites or regions annotated in the human genome. Here, mutations in splice sites and regions were significantly enriched in aSHM motifs, but only if the mutations were proximal to a TSS (AID target regions), which is consistent with our hypothesis and previous observations [4] (Fisher’s exact test, splice sites *p* < 0.01, splice regions *p* < 0.0001; Fig. 2A). Complementarily, we used as a second background distribution the aSHM/non-aSHM contexts of all proximal intronic mutations, which we assumed to be under neutral evolution. We found that only donor sites and conserved donor regions had a significant enrichment in proximal RCH/TW mutations among the tested genomic features (Fisher’s exact test, donor sites odds ratio (OR) = 3.39, conserved donor regions OR = 2.44, *p* < 0.0001; Fig. 2B).

We tested if our findings could be extrapolated to (1) other DLBCL cohorts; and (2) cohorts of cancers without AID activity. For DLBCL, we used the recurrent splice site mutations in our WXS meta-cohort of > 1,800 DLBCLs [15] and WGS data from MALY-DE. For other cancers, we selected datasets from the ICGC project corresponding to 12 different cancer types for which AID-associated mutational signatures seem to be absent [5, 21] (Additional file 1). Because some datasets were WXS, we could not use intronic mutations as a reliable background, and instead, we used the motif distribution of all genomic splice sites. We found enrichment in proximal RCH/TW splice site mutations in all DLBCL cohorts (Fisher’s exact test, *p* < 0.01; Fig. 2C), but not in any of the cancer types without AID activity. Again, this enrichment was not observed in regions distal to TSSs, out of the working range of AID activity. The chronic lymphocytic leukemia (CLL) cohort has been described to have AID activity in ≈30% of the samples [21], which may explain the lack of significant enrichment in RCH/TW splice site mutations in our analysis.

To further test if AID preferentially mutates splice sites, we reanalyzed germinal center B-cells sequencing data from *Aicda*^*−/−*^ and from *Ung*^*−/−*^*Msh2*^*−/−*^ mice from Alvarez-Prado et al. [16]. The *Ung*/*Msh2* double knockout forces all the C > U deaminations caused by AID to be corrected to T by the replication process, making this model ideal to reveal AID-driven mutations. We found conserved donor regions and donor sites to be the top genomic features enriched in C > T transitions associated with AID activity (Fisher’s exact test, donor regions OR = 3.43, donor sites OR = 3.05, *p* < 0.0001; Fig. 2D). These results on mouse models confirmed that AID preferentially mutates splice sequences over other gene regions.

Finally, in order to assess the impact of AID-caused splice site mutations in DLBCL clonal diversity, we analyzed the estimated cancer cell fraction (CCF) of each splice site variant from Chapuy et al. cohort [6], which represents the fraction of cancer cells in each sample containing the mutation. We observed that 74.70% (62/83) of splice site mutations in potential AID targets are clonal (CCF ≥ 0.9), whereas splice site mutations in non-AID trinucleotide contexts or in distal RCH/TW motifs present lower percentages of clonality (non-AID, proximal: 63.33%; AID, distal: 57.79%, non-AID, distal: 55.32%; Fig. 2E). The CCF of a mutation can be used as a surrogate measure of the time of acquisition, as it is assumed that clonal alterations occur before subclonal ones [22]. This implies that splice site mutations caused by AID, which are mostly clonal, are earlier driver events than other, non-related to aSHM, splice site variants in DLBCL. Therefore, we can conclude that splice site mutations caused by AID potentially yield relevant loss-of-function of several genes at the onset of lymphoma.

## Conclusion

In conclusion, aSHM causes recurrent clonal splicing mutations in DLBCL due to the high conservation of RCH and TW motifs in these genomic regions. As a result, these mutations are expected to alter the function of several proteins, some of them (like in CD79B [15] or BCL7A [19]) being positively selected in the lymphoma context.

### Supplementary Information

**Supplementary Material 1.**

**Supplementary Material 2.**

## Data Availability

All the datasets analyzed in this study are publicly available. Information on each dataset access is detailed in Additional file 1.

## Abbreviations

DLBCL: Diffuse large B-cell lymphoma
SHM: Somatic hypermutation
aSHM: Aberrant somatic hypermutation
AID: Activation-induced cytidine deaminase
WGS: Whole genome sequencing
WXS: Whole exome sequencing
TSS: Transcription start site
UNG: Uracil-DNA glycosilase
MSH2/6: MutS homolog 2/6
OR: Odds ratio
ICGC: International Cancer Genome Consortium
CLL: Chronic lymphocytic leukemia
FL: Follicular lymphoma
CNS: Central nervous system
SCC: Squamous cell carcinoma
C: Conserved
NC: Non-conserved
CDS: Coding sequence
UTR: Untranslated region
FDR: False discovery rate
ns: Non-significant
CCF: Cancer cell fraction
