# Supplementary material for: Activation-induced cytidine deaminase causes recurrent splicing mutations in diffuse large B-cell lymphoma
Source: Mol Cancer. 2024 Feb 24;23:42. doi: 10.1186/s12943-024-01960-w (PMC10893679; doi:10.1186/s12943-024-01960-w)
Supplement: Supplementary file 2 — Supplementary Material 2. [file 12943_2024_1960_MOESM2_ESM.docx]

**Supplemental Material: Activation-induced cytidine deaminase causes recurrent splicing mutations in diffuse large B-cell lymphoma**

Benitez-Cantos et al.

# Table of contents

[Table of contents 1](#_Toc149918366)

[Supplemental Methods 2](#_Toc149918367)

[Data and annotation resources 2](#_Toc149918368)

[AID mutagenesis enrichment analysis 2](#_Toc149918369)

[*Aicda*^-/-^ and *Ung/Msh2*^-/-^ mice data analysis 2](#_Toc149918370)

[Supplemental File Descriptions 4](#_Toc149918371)

[References 5](#_Toc149918372)

#

# Supplemental Methods

## Data and annotation resources

DLBCL somatic mutations from Andrades et al (1) and Arthur et al (2) were retrieved from the supplementary information of the manuscripts. Somatic mutations from International Cancer Genome Consortium (ICGC) cohorts were retrieved from the ICGC Data Portal (<https://dcc.icgc.org/search>. Accessed 13 January 2023). Presence or absence of AID mutational signatures per cancer type was reported in previous publications (3,4) (**Additional File 1**). We considered SBS84 (RCH-like), SBS9 and SBS85 (TW-like) as AID-associated signatures, according to their proposed aetiology in COSMIC Mutational Signatures (v3.3 - June 2022). Two ICGC cohorts without AID activity (KICH-US and CMDI-UK) were excluded from the analysis due to a low number of splice site mutations. LAML-US was excluded as the data of only 9 donors was used for mutational signature discovery (3,4). 41 MALY-DE samples were filtered out as they were included in the Arthur et al cohort. Unless otherwise specified, all analyses were performed using GENCODE v19 (Ensembl 74) as an annotation resource, and GRCh37/hg19 as the reference human genome. Mis-splicing intronic mutations were retrieved from Tables S2 and S3 from Jung et al (5) and cancer cell fraction (CCF) data were obtained from Chapuy et al (6) supplementary material (Table S3D).

We reannotated the mutations in protein coding genes with the following genomic features adapted from the variant consequences established by Ensembl (<https://www.ensembl.org/info/genome/variation/prediction/predicted_data.html>): splice acceptor (2-base region at the 3’ end of an intron), splice donor (2-base region at the 5’ end of an intron), CDS, conserved donor region (positions +3 to +5 at the 5’ end of an intron), non-conserved donor region (positions +6 to +8 at the 5’ end of an intron), acceptor region (positions -3 to -8 at the 3’ end of an intron), 5’ UTR, 3’ UTR, intron.

## AID mutagenesis enrichment analysis

We considered that a mutation follows an aSHM pattern if it is a single base substitution in RCH or TW contexts (or their reverse complementary counterparts) within <3 kb from the TSS of protein coding genes. For each cancer dataset, we calculated the number of AID / non-AID mutations and compared this distribution to either intronic mutations from the same dataset (for WGS data) or the distribution of AID / non-AID contexts in the reference genome (for WGS or WES) for a given genomic feature (e.g. splice donors, CDS or 5’UTR). Statistical significance of differences between distributions was assessed with Fisher’s exact test in R (version 3.6.3). P values were FDR-corrected with the p.adjust() function.

## *Aicda*^-/-^ and *Ung/Msh2*^-/-^ mice data analysis

Targeted sequencing FASTQ files generated by Álvarez-Prado et al (7) were obtained from the NCBI Sequence Read Archive for one *Aicda*^-/-^ mouse (SRR5956203) and two *Ung*^-/-^*Msh2*^-/-^ mice (SRR5956198, SRR5956202). The authors sequenced 1588 genomic regions corresponding to the first 500 bp downstream of each transcriptional start site of 1379 genes. Reads were aligned to the mouse genome GRCm38 (downloaded from ftp://[ftp.ncbi.nlm.nih.gov/genomes/all/GCA/000/001/635/GCA_000001635.8_GRCm38.p6/](http://ftp.ncbi.nlm.nih.gov/genomes/all/GCA/000/001/635/GCA_000001635.8_GRCm38.p6/)) using bwa-mem (version 0.7.17). Sorted BAM files were transformed into pileup format with samtools mpileup (version 1.7) and coverage and transitions per nucleotide were calculated, as well as transition frequency (# transitions / coverage). C>T and G>A changes were only considered transitions if the Phred score was > 15. Then, positions outside of the amplicons specified in the supplementary information of Álvarez-Prado et al (7), converted to GRCm38 with the liftOver R package (R version 3.6.3, Bioconductor version 3.9.0), and single nucleotide polymorphisms annotated in the Sanger Mouse Genomes Project SNP and Indel (release v5) were excluded from the analysis. The resulting coordinates were annotated with GENCODE vM25 to calculate the transition enrichment for the aforementioned genomic features (Fisher’s exact test).

#

# Additional File Descriptions

**Additional File 1**. Metadata of the 16 datasets analyzed in this study including data sources and AID mutational signatures information.

# References

1. Andrades A, Álvarez-Pérez JC, Patiño-Mercau JR, Cuadros M, Baliñas-Gavira C, Medina PP. Recurrent splice site mutations affect key diffuse large B-cell lymphoma genes. Blood. 2022 Apr 14;139(15):2406–10.

2. Arthur SE, Jiang A, Grande BM, Alcaide M, Cojocaru R, Rushton CK, et al. Genome-wide discovery of somatic regulatory variants in diffuse large B-cell lymphoma. Nat Commun. 2018 Dec;9(1):4001.

3. Alexandrov LB, Kim J, Haradhvala NJ, Huang MN, Tian Ng AW, Wu Y, et al. The repertoire of mutational signatures in human cancer. Nature. 2020 Feb 6;578(7793):94–101.

4. Bergstrom EN, Luebeck J, Petljak M, Khandekar A, Barnes M, Zhang T, et al. Mapping clustered mutations in cancer reveals APOBEC3 mutagenesis of ecDNA. Nature. 2022 Feb 17;602(7897):510–7.

5. Jung H, Lee KS, Choi JK. Comprehensive characterisation of intronic mis-splicing mutations in human cancers. Oncogene. 2021 Feb 18;40(7):1347–61.

6. Chapuy B, Stewart C, Dunford AJ, Kim J, Kamburov A, Redd RA, et al. Molecular subtypes of diffuse large B cell lymphoma are associated with distinct pathogenic mechanisms and outcomes. Nat Med. 2018 May;24(5):679–90.

7. Álvarez-Prado ÁF, Pérez-Durán P, Pérez-García A, Benguria A, Torroja C, de Yébenes VG, et al. A broad atlas of somatic hypermutation allows prediction of activation-induced deaminase targets. J Exp Med. 2018 Mar 5;215(3):761–71.
